# Clear Cell Renal Cell Carcinoma: A Test Bench for Investigating Tumor Complexity

**DOI:** 10.3390/cancers16040829

**Published:** 2024-02-19

**Authors:** Claudia Manini, Estíbaliz López-Fernández, Gorka Larrinaga, José I. López

**Affiliations:** 1 Department of Pathology, San Giovanni Bosco Hospital, ASL Città di Torino, 10154 Turin, Italy; claudia.manini@aslcittaditorino.it; 2 Department of Sciences of Public Health and Pediatrics, University of Turin, 10124 Turin, Italy; 3 FISABIO Foundation, 46020 Valencia, Spain; estibaliz.lopez@universidadeuropea.es; 4 Faculty of Health Sciences, European University of Valencia, 46023 Valencia, Spain; 5 Department of Nursing, Faculty of Medicine and Nursing, University of the Basque Country (UPV/EHU), 48940 Leioa, Spain; gorka.larrinaga@ehu.eus; 6 Department of Physiology, Faculty of Medicine and Nursing, University of the Basque Country (UPV/EHU), 48940 Leioa, Spain; 7 Biobizkaia Health Research Institute, 48903 Barakaldo, Spain

Clear cell renal cell carcinoma (CCRCC), by far the most common renal cancer subtype, is an aggressive tumor variant, serving in recent years as a prolific test bench in cancer research. Many of the recent advances in our knowledge about intratumor heterogeneity, tumor evolution, eco-oncology, metastatic competence, and therapeutic resistance have been achieved by analyzing this neoplasm [1,2,3]. The varied spectrum of papers collected in this Special Issue, composed of five articles, six reviews, one systematic review, and one commentary, confirms this fact.

Here, basic researchers and clinicians from Italy, Japan, USA, Spain, South Africa, Sweden, Romania, and Germany present their findings from very different perspectives, i.e., the role of the complement system in the immune background of tumors [4], imaging-based biomarker identification [5], the relevance of the immune microenvironment [6,7], the influence of clock genes and the circadian rhythm in tumor therapy [8], the morphological/molecular characteristics of cystic CCRCCs [9], the single-cell RNA-sequencing signature of primary and metastatic neoplasms [10], an extensive review of tumor biomarkers in CCRCC [11], the nitric oxide cycle-related pathways of this tumor [12], the current trends and complications of partial and radical nephrectomy in CCRCC [13], the characteristics of the intratumor immune heterogeneity in non-metastatic tumors [14], the importance of NPC1 targeting in CCRCC [15], and a comparison between the efficacy of immune checkpoint inhibitors (ICI) and tyrosine kinase inhibitors (TKI)/everolimus in the adjuvant therapy of CCRCC [16].

Panebianco et al. [4] review the role of the complement system (CS) in fostering the growth and progression of CCRCC through its interaction with the tumor microenvironment. The authors detail first the physiology of the CS, including the canonical and non-canonical pathways of complement activation and its negative regulators. Then, they analyze the role of the CS in cancer in general and in CCRCC in particular. Finally, the CS as a possible target in cancer therapy is considered. They conclude that CS may represent a predictive marker in the evaluation of immune checkpoint inhibitors-based therapies.

Posada Calderon et al. [5] focus on the difficulties of distinguishing renal cell carcinoma from other renal diseases based on current techniques of imaging. Magnetic resonance imaging (MRI) and positron emission tomography (PET)/CT using different radiolabeled molecules such as ^18^F-fluorodeoxyglucose, ^124^I-cG250, radiolabeled prostate-specific membrane antigen, and ^11^C-acetate, together with a computational approach to CT images, are unveiling some data; however, in the opinion of the authors, these data still require standardization and external validation before their integration into clinical practice. In this sense, recent research proposes two new feature selection strategies in the interpretation of radiomics studies to predict molecular and clinical targets in CCRCC [17].

In their first contribution to this Special Issue, Shapiro et al. [7] review the complexity of the immune microenvironment in renal cell carcinomas with special focus on CCRCC. The authors contextualize the issue starting with the immune response to cancer in general and the cells involved in the tumor immune microenvironment. Then, they follow with the characteristics of the immune environment in renal cancer, paying special attention to the different lymphocyte subsets involved and to tumor-associated macrophages. The role of tumor neoantigens and the genomic correlations with the immune response, as well as the predictive and prognostic ability of the immune microenvironment in CCRCC survival, have been also considered in their review. They conclude by emphasizing the critical importance of the tumor immune microenvironment in the understanding of the still hidden mechanisms operating in this neoplasm.

CCRCC exhibits resistance to standard chemotherapy, and the objective of current first-line therapies is to control tumor angiogenesis via tyrosine TKIs and to inhibit immune checkpoints through ICIs [18], which indicates the importance of the tumor microenvironment (TME) in the intricate biology of the CCRCC ecosystem. The interplay between cancer-associated fibroblasts (CAF), a predominant constituent of tumor stroma, and immune cells in the TME emerges as a decisive factor in CCRCC progression and in therapeutic responsiveness [19,20,21]. Through cytokine secretion, CAFs have the capability to induce the differentiation of M2 macrophages and attenuate the cytotoxic capacity of CD8 cells, fostering an immunosuppressive milieu [19,22]. In addition, CAFs’ remodeling actions on the extracellular matrix impede the arrival of tumor-killing immune cells [22]. CAFs exhibit abundant expression of fibroblast activation protein-α (FAP) [23], a key player in these biological phenomena [22]. Both mRNA and immunohistochemical FAP expression in CAFs correlate with poor prognosis and limited response to TKIs and ICIs [20,21,24]. Current evidence suggests that FAP is a promising biomarker and a target for histopathological imaging, advanced radio-diagnostics, and emerging therapeutic modalities [25]. As a recent example of diagnostic usefulness, FAP+ CAFs specifically concentrate at the point of stromal invasion in colonic biopsies with uncertain invasion, thus helping in diagnosing adenocarcinoma microinvasion [26].

In a very interesting article, Fujita et al. [6] analyze the association of the tumor immune microenvironment in the primary tumor with the intervals of metastases. For this purpose, the authors considered synchronous (metastases within 3 months) and metachronous (metastases after 3 months) CCRCC in a series of 568 patients. They found that PD-L1 expression in tumor-infiltrating immune cells and immunophenotypes of the primary tumor were different according to the time recurrence, with the increased PD-L1 expression and the inflamed phenotypes being associated with shorter recurrences and tumor aggressiveness. These findings agree with previous experiences with inflamed [27] and PD-L1+ [28] CCRCC.

The circadian rhythm is involved in the regulation of cellular differentiation and physiology. Santoni et al. [8] revisit the influence of the altered expression of clock genes on the onset of cancer. The authors first consider the role of clock genes in cancer focusing specifically on renal cell carcinoma tumorigenesis and prognosis. They also analyze the circadian variations in cytokines and chemokines and their influence on the efficacy of immunotherapy and targeted therapy. They conclude that the role of the circadian clock genes in patients with renal cell carcinoma deserves further investigation since they represent potential therapeutic targets.

Pini et al. [9] review the morphological and molecular characteristics of cystic clear cell renal cell carcinomas. They point specifically to CCRCC with cystic changes, multilocular CCRCC, and clear cell papillary renal cell carcinomas. This short review provides useful criteria for pathologists when making a differential diagnosis in daily practice. The review includes a complete list of references on this topic.

A review of the recent advances in single-cell RNA-sequencing of CCRCC (primary and metastatic) [10] shows the importance of new tools in unveiling cancer biology. The authors revisit the origin of tumor cells in CCRCC, the transcriptomic identity of metastasizing cells, the role of the tumor microenvironment, immune and non-immune, in cancer progression, and the treatment opportunities of metastatic and non-metastatic CCRCCs.

Since CCRCC is a paradigm of intratumor heterogeneity, the selection of the best treatment modality in every CCRCC patient is a difficult task. In a systematic review, Dani et al. [11] present valuable insights into the spectrum of biomarkers available in predicting treatment response, prognosis, and therapeutic monitoring in patients with metastatic disease. The authors revisit retrospective studies and analyze prospective options in immunotherapy, VEGF-TKIs, and mTOR treatment strategies.

Ene et al. [12] analyze the role of the nitric oxide (NO) in the therapy of CCRCC. The authors review fundamental aspects such as the disruption of NO homeostasis, the dysregulation ureagenic cycle, the upregulation of glutamine, the cellular depletion of arginine, hyperammonemia, the reduction in branched-chain amino acids, the inactivation of VHL and the accumulation of HIFs, and the endogenous inhibition of NO synthesis.

Surgical trends and complications in partial and radical nephrectomy are reviewed by Pyrgidis et al. [13], using the GeRmAn Nationwide Inpatient Data provided by the Research Data Center of the Federal Bureau of Statistics of Germany between 2005 and 2021. The authors compare the perioperative morbidity, mortality, hospital stay, and costs between patients who underwent partial and radical nephrectomies. They show that the number of partial nephrectomies increased in this period. The authors detect an increment in comorbidities and risk factors in patients selected for radical nephrectomy. For example, statistically significant differences were detected in several clinical complications, such as transfusion, sepsis, acute respiratory failure, acute kidney disease, thromboembolism, ileus, surgical wound infection, 30-day mortality, and intensive care unit admission, between the two groups.

The second contribution of Shapiro et al. [14] points to the immune cell infiltration heterogeneity in non-metastatic CCRCC. The authors find that an increased number of CD8 cells within the tumor is associated with a decreased likelihood of progression to metastatic disease. In addition, they find that CD8 cells situated in close proximity to tumor cells are a sign of non-metastatic evolution in these patients. These findings strengthen the role of CD8 cells as a prognostic biomarker in CCRCC.

Proliferating cancer cells have greater requirements for cholesterol than non-tumor cells, and Fazliyeva et al. [15] show in their study that CCRCC cells have redundant mechanisms of cholesterol acquisition; for example, all major lipoproteins have comparable ability to support tumor cell growth and are equally effective in counteracting the antitumor activity of TKIs. Interestingly, the endolysosomal cholesterol transport regulated by the Niemann–Pick type C1 (NPC1) protein is a therapeutic target because this is a point where lipoproteins-derived cholesterol trafficking routes converge and may be simultaneously targeted in CCRCC patients.

Ossato et al. [16] aim in their study to compare the efficacy of mTOR, TKI, and CI inhibition therapies in metastatic CCRCC using a reconstruction of individual patient data from Kaplan–Meier curves. This novel approach allowed the authors to conduct all indirect head-to-head comparisons between these agents in a context in which no ‘real’ comparative trials have been conducted.

To conclude, this Special Issue comprises the recent experience of basic researchers and clinicians in several key issues of CCRCC pathogenesis, evolution, and treatment modalities that currently impact patient prognosis. Once more, the convenience of promoting multidisciplinary and translational approaches in cancer research is highlighted using CCRCC as a test bench.

## Data Availability

Not applicable.
